# Immunoinformatic analysis for identifying immunogenic antigens from the complete proteome of *Giardia lamblia*

**DOI:** 10.1590/0074-02760250216

**Published:** 2026-04-17

**Authors:** David Ortega-Tirado, Carlos A Velazquez-Valdez, Thania Garzon, Leslie Bracamontes-Picos, Gloria Lopez-Romero, Carlos Velazquez

**Affiliations:** 1 Universidad de Sonora, Department of Chemistry-Biology, Hermosillo, Sonora, Mexico; 2 Universidad de Sonora, Department of Mathematics, Hermosillo, Sonora, Mexico

**Keywords:** T cell epitopes, Giardia lamblia, immunogenic polypeptide, MHC-II

## Abstract

**BACKGROUND:**

*Giardia lamblia* is a parasite that infects humans. To date, there is no vaccine available for human giardiasis. Thus, discovering new immunogenic antigens is crucial for the rational design of a vaccine.

**OBJECTIVES:**

This study aimed to identify the main immunogenic antigens of *G. lamblia* from its entire proteome using immunoinformatic and data science techniques. To our knowledge, this is the first study to systematically identify immunogenic antigens of *G. lamblia* across its complete proteome, providing a comprehensive map of potential immunogenic antigens.

**METHODS:**

Briefly, FASTA sequences of *G. lamblia* isolates WB and GS were submitted to the NetMHCII 2.3 predictor. The analysis was conducted for five murine major histocompatibility complex (MHC)-II molecules: I-Ab, I-Ad, I-Ed, I-Ak, and I-Ek. Python 3.9 was used to develop custom code for data processing and analysis.

**FINDINGS:**

We identified 414 potential immunogenic polypeptides for isolate WB and 350 for isolate GS. For both isolates, most polypeptides contained peptides with high affinity for I-Ab and I-Ek. Notably, no polypeptides with high affinity for I-Ak were detected. Homologous potential immunogenic antigens (129 polypeptides) were identified in both isolates. The analysis revealed that 12 potential immunogenic polypeptides from isolate WB and 10 from isolate GS are part of the *Giardia* secretome. Additionally, promiscuous polypeptides that bind to at least two different MHC-II molecules were found in both isolates.

**MAIN CONCLUSIONS:**

These findings lay a valuable foundation for the rational development of a vaccine against human giardiasis and show a computational strategy that can be applied to the study of other pathogens.

*Giardia lamblia* is a protozoan parasite that infects the upper intestinal tract of humans and other mammals.^1^ This parasite has a life cycle consisting of an infective form known as a cyst and a vegetative form called a trophozoite.^2^ ^,^ ^3^ Infection begins when a host ingests water or food contaminated with cysts. Once in the stomach, the excystation process begins, and each cyst generates two trophozoites. The trophozoites colonise the small intestine without invading the epithelia. Eventually, they migrate to the lower intestinal tract, where environmental changes induce the encystation process, and the cysts are then released through the faeces, completing the life cycle.^2^ ^,^ ^3^

*Giardia lamblia* is divided into eight assemblages, A-H.^4^ ^,^ ^5^ Assemblages A and B are the main ones responsible for human giardiasis.^4^ ^,^ ^5^ To date, there is no vaccine against human giardiasis. The discovery of new immunogenic antigens of *Giardia* therefore remains pivotal for the rational design of a vaccine against this disease.

Several *Giardia* antigens have confirmed immunogenicity. Some of these are structural molecules, such as α-giardins and α- and β-tubulins.^6^ Metabolic enzymes, including fructose-1,6-bisphosphate aldolase (FBA), ornithine carbamoyltransferase (OCT), arginine deiminase (ADI), and enolase, are also sources of immunogenic and conserved molecules.^7^ Surface antigens, predominantly variant-specific surface proteins (VSPs), constitute a group of *Giardia* proteins with high immunogenic properties.^8^ Even in the cyst stage, immunogenic molecules such as cyst wall proteins CWP1, CWP2, and CWP3 are present. In addition, the excretory-secretory products of *Giardia*, which shape the parasite’s secretome, contain immunogenic proteins, including cathepsins, VSPs, metabolic enzymes such as ADI, OCT, and enolase, and structural proteins such as α-1 giardin, α-2 giardin, and α-11 giardin.^6^ ^,^ ^9^ These *Giardia* antigens can trigger the immune response necessary to control infection.

It is well established that the adaptive immune response is essential not only for controlling but also for clearing *Giardia* infection. CD4^+^ T cells are pivotal for parasite elimination, as previous studies have shown that CD4^+^-deficient mice are unable to clear *Giardia* infection.^10^ These cells are also relevant in human parasitic infections, as observed in patients with HIV infection. Studies have revealed a higher prevalence of parasites such as *Cryptosporidium* spp. and *G. lamblia* among human immunodeficiency virus (HIV)-infected children with low CD4^+^ T-cell counts.^11^ Similarly, in adult patients with HIV infection, *G. lamblia* is the most frequently identified parasite, followed by *Entamoeba histolytica* and *Ascaris lumbricoides*.^12^ ^,^ ^13^ Accordingly, the search for new immunogenic antigens could be directed towards those capable of activating CD4^+^ T cells.

Despite progress in the identification of immunogenic antigens of this parasite, our understanding of the full repertoire of potential immunogenic proteins encoded in the genome of *G. lamblia* remains limited. The classical route for antigen discovery begins with the cultivation of the target pathogen under laboratory conditions, followed by its dissection into individual components, the identification and isolation of each component, and the evaluation of the immunogenic capacity of the antigens.^14^ ^,^ ^15^ Subsequently, selected antigens are produced on a large scale to initiate vaccine development. This classical workflow is costly and time-consuming and may also pose biological hazards due to pathogen manipulation and culture.

Nowadays, immunoinformatics represents a faster and more accessible approach for uncovering immunogenic proteins. This technique starts from the pathogen’s genome and combines various computational tools to identify immunogenic proteins based on the recognition of peptides by B and/or T cells.^15^ For the identification of peptides recognised by CD4^+^ T cells, different prediction algorithms are used to identify major histocompatibility complex (MHC)-binding sequences within protein antigens. This approach serves as an alternative strategy for the discovery of new immunogenic antigens in *Giardia* and other pathogens.

Based on the above considerations, the present study aimed to apply immunoinformatics approaches to identify all potential immunogenic proteins from the complete proteome of *G. lamblia*. Selection was based on the identification of T-cell peptides with affinity for MHC class II molecules using the NetMHCII 2.3 tool from the immune epitope database (IEDB). Python 3.9 was also used to process and analyse all derived data. The source of the *G. lamblia* proteomes was GiardiaDB, a repository containing genome and proteome data from several *Giardia* genotypes, encompassing all proteins associated with this parasite. Some data have been validated using microarrays and mass spectrometry, and deprecated genes have been removed.^16^ GiardiaDB also contains computationally predicted protein sequences, which may include redundancies or unvalidated entries, thereby highlighting the potential for further investigation.

## MATERIALS AND METHODS

*Data acquisition* - FASTA files containing the full proteomes of *G. lamblia* isolates WB and GS were downloaded from the GiardiaDB repository^17^ (https://giardiadb.org/giardiadb/app). In addition, XLSX files containing general information on both proteomes were obtained, including amino acid length, molecular weight, the presence of transmembrane domains, and whether the polypeptides are associated with the trophozoite or cyst stage. It is worth noting that GiardiaDB includes computationally predicted protein sequences, which provides opportunities for further validation in downstream analyses.

*MHC-II peptide binding prediction* - The analysis was conducted between February 2023 and January 2024. Predictions were performed using the NetMHCII 2.3 tool, accessible via the IEDB (www.iedb.org) RESTful API.^18^ ^,^ ^19^ Implementation was carried out using the Python SDK for the IEDB API Tools to facilitate interaction with the API for peptide-binding prediction. Predictions were performed against five murine MHC class II molecules: I-Ak, I-Ek, I-Ad, I-Ed and I-Ab. The output from the MHC class II peptide-binding predictions was merged with general polypeptide information. This integration enabled a comprehensive analysis, combining binding affinity predictions with general features of each polypeptide. A polypeptide was considered immunogenic if at least one of its T-cell peptides bound to any of the MHC class II molecules described above. Once the data were merged, a general analysis was conducted for the different percentile ranks available. This step provided an overview of peptide-binding affinities across the proteome, allowing the identification of polypeptides with high binding potential. For a more precise analysis, the data were subsequently filtered to include only polypeptides with T-cell peptides exhibiting a percentile rank ≤ 0.01, an IC_50_ value ≤ 50 nM, and a protein length ≤ 3,000 amino acids. These filtering criteria ensured the selection of polypeptides with the highest binding affinity within biologically relevant polypeptide length limits.

*Identification of immunogenic polypeptides in the secretome of G. lamblia assemblages A and B* - To identify all immunogenic polypeptides in the secretomes of the GS and WB isolates of *G. lamblia*, the filtered MHC class II prediction data were merged with the XLSX files obtained from the study by Ma’ayeh and colleagues.^9^ The XLSX files used for the merge were titled: “S1 Table. All proteins identified in the secretome of Giardia intestinalis WB isolate in serum-free RPMI-1640 medium” and “S2 Table. All proteins identified in the secretome of Giardia intestinalis GS isolate in serum-free RPMI-1640 medium”.

*Homology analysis of the immunogenic polypeptides of G. lamblia* - The filtered MHC class II prediction data were subjected to homology analysis. Specifically, polypeptides from *Giardia* Assemblage B isolate GS and *Giardia* Assemblage A isolate WB were analysed to identify those conserved in both isolates. Sequence similarity was calculated using the Biopython module, which performs pairwise sequence alignment via a dynamic programming algorithm. Polypeptides with a sequence identity ≥ 80% were considered homologous.

*Analysis of the interactions of the immunogenic T-cell peptides of G. lamblia with MHC-II molecules* - We conducted a literature search to identify the structures of the MHC class II molecules analysed in this study: I-Ad, I-Ek, and I-Ak.^20^ ^,^ ^21^ ^,^ ^22^ The anchor residues of model peptides bound to I-Ad, I-Ek, and I-Ak were compared with the most immunogenic T-cell peptides from the WB and GS isolates. Based on the filtered MHC class II binding prediction data, T-cell peptides with the lowest IC_50_ values were selected as the most immunogenic for both *G. lamblia* isolates.

## RESULTS

*Immunogenic polypeptides identification by using immunoinformatic and the whole G. lamblia proteome* - The rational design of vaccines involves the identification of immunogenic antigens of the pathogen. In this study, our objective was to identify immunogenic polypeptides from the entire proteome of *G. lamblia*. We used the proteomes of the WB isolate (assemblage A) and GS isolate (assemblage B) from GiardiaDB to predict T-cell peptides with the highest affinity for MHC class II molecules. Out of 4,970 polypeptide chains (representing 100% of the *G. lamblia* WB proteome) submitted for prediction, a total of 414 chains (8%) were identified as immunogenic (percentile rank ≤ 0.01 and IC_50_ ≤ 50 nM) for the WB isolate (Figure A). Of these, 33% were associated with I-Ek, 31% with I-Ab, 23% with I-Ad, and 13% with I-Ed. No immunogenic polypeptides were identified for I-Ak under the selected parameters.

Out of a total of 4,470 polypeptide sequences (representing 100% of the GS isolate proteome), 350 chains (8%) were identified as immunogenic (Figure B). Among these, 36% of the polypeptides were bound to I-Ek, 31% to I-Ab, 17% to I-Ad, and 16% to I-Ed. No immunogenic polypeptides with affinity for I-Ak were identified under the selected parameters (percentile rank ≤ 0.01 and IC_50_ ≤ 50 nM).

The designation of a polypeptide as immunogenic was based on its content of T-cell peptides with affinity for MHC class II molecules. Accordingly, we also report the number of T-cell peptides per polypeptide. For the WB isolate, 1,402 T-cell peptides were identified (100%), of which 34% showed affinity for I-Ek, 31% for I-Ab, 21% for I-Ad, and 14% for I-Ed (Figure C). For the GS isolate, 1,231 T-cell peptides were identified (100%), with 36% showing affinity for I-Ek, 31% for I-Ab, and 16% each for I-Ad and I-Ed (Figure D). No peptides with affinity for I-Ak were identified under the selected parameters for either isolate.

**Figure:**
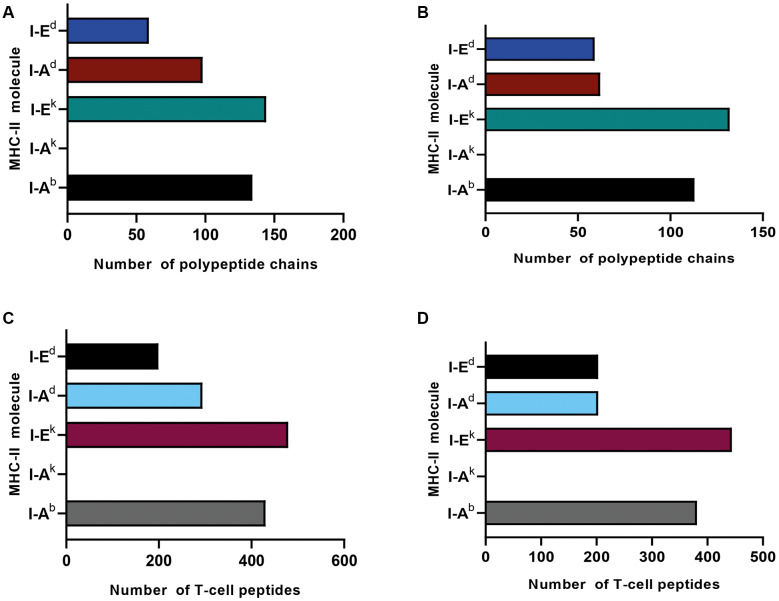
The *Giardia lamblia* proteome contains immunogenic polypeptides and T-cell peptides that predominantly interact with I-Ab and I-Ek molecules. The *Giardia* proteome was obtained from GiardiaDB and analysed using NetMHCII 2.3 via immune epitope database (IEDB). A Python 3.9 script was developed to process the data. The analysis included five murine major histocompatibility complex (MHC) class II molecules: I-Ed, I-Ad, I-Ek, I-Ak, and I-Ab. The graphics show the distribution of polypeptides and T-cell peptides for each MHC class II molecule. Panels A and C correspond to *Giardia* assemblage A isolate WB, while panels B and D correspond to *Giardia* assemblage B isolate GS.

We also performed a homology analysis to identify immunogenic polypeptides with similar amino acid sequences between the WB and GS isolates [Supplementary data (Table I)]. The amino acid sequences of the 414 polypeptide chains identified from the WB isolate were compared with the 350 chains from the GS isolate. A total of 129 polypeptides exhibited ≥ 80% sequence homology. The T-cell peptides from each polypeptide are presented, highlighting that each pair of homologous polypeptide chains shares the same peptide core.

Antigen presentation can be enhanced when peptides are capable of binding to more than one MHC molecule. Our results showed that 21 polypeptide chains out of 414 in the WB isolate contained T-cell peptides that could be presented by multiple MHC class II molecules [Supplementary data (Table II)]. These molecules are referred to as promiscuous polypeptide chains. Sixteen promiscuous polypeptide chains were also identified in the GS isolate [Supplementary data (Table III)].

*The immunogenic proteins of the secretome of G. lamblia* - *G. lamblia* is unable to cross the intestinal epithelial barrier, which raises questions about how this protozoan triggers an adaptive immune response. One possible explanation involves the excretory-secretory products of *G. lamblia*, which constitute the *Giardia* secretome. We therefore investigated whether any of the identified immunogenic polypeptides were also present in the *Giardia* secretome. A comparative analysis was performed between the immunogenic polypeptides identified in this study and the molecules detected in the secretomes of *G. lamblia* isolates WB and GS.^9^ For the WB isolate, 12 immunogenic polypeptides (3% of 414) were found to belong to the secretome (Table I). These antigens include cathepsins, high-cysteine membrane proteins, and enzymes involved in glycolysis. The T-cell peptides generated from each polypeptide are also listed, with the peptide core sequence shown; the core is identical for all peptides derived from the same polypeptide.

**TABLE I:** Immunogenic polypeptides present in the secretome of *Giardia* assemblage A isolate WB

Polypeptide ID	Polypeptide name	TM domains	Polypeptide length	Molecular weight (kDa)	T-cell peptides	MHC-II
GL50803_006330	Unspecified product	No	271	29	194KW YSYHAALAT DLLL208, 192ATKWY SYHAALAT DL206, 193TKW YSYHAALAT DLL207	I-Ab
GL50803_0016779 ^1^	Cathepsin B	No	298	33	6LAA AAFSAPALT VSE20, 5LLAA AAFSAPALT VS19, 7AA AAFSAPALT VSEL21, 8A AAFSAPALT VSELN22, 3LFLLAA AAFSAPALT 17, 4FLLAA AAFSAPALT V18	I-Ab
GL50803_0014019 ^4^	Cathepsin B	No	300	33	8A AAFSAPALT VSELN22, 3LFLLAA AAFSAPALT 17, 4FLLAA AAFSAPALT V18, 7AA AAFSAPALT VSEL21, 6LAA AAFSAPALT VSE20, 5LLAA AAFSAPALT VS19	I-Ab
GL50803_002834	Putative GTPase activating protein for ARF	No	398	42	211QTAP AMFTAPAVS TM225, 213AP AMFTAPAVS TMPA227, 212TAP AMFTAPAVS TMP226	I-Ab
GL50803_009117 ^2^	cAMP-dependent protein kinase regulatory chain	No	460	51	46IHK FASYSPLAA AVL60	I-Ab
GL50803_008528	Unspecified product	No	528	57	236FDVL VTFQPAMAA KL250	I-Ab
GL50803_0027717	High cysteine membrane protein Group 3	Yes	836	86	245GRCI SAFSAASAL AG259, 246RCI SAFSAASAL AGC260, 247CI SAFSAASAL AGCS261, 248I SAFSAASAL AGCST262, 249SA FSAASALAG CSTY263	I-Ab
GL50803_0013922	Unspecified product	Yes	1087	114	238V FRYSVASAA LVKGK252, 234YQNAV FRYSVASAA L248, 237AV FRYSVASAA LVKG251, 235QNAV FRYSVASAA LV249, 236NAV FRYSVASAA LVK250	I-Ab
GL50803_00102101	Kinesin-3	No	1026	115	728KF LRKYSALRA LFSH742	I-Ad
GL50803_0016125 ^3^	FAD-dependent glycerol-3-phosphate dehydrogenase	No	1111	119	358IM YSFASARAV LPSN372, 359M YSFASARAV LPSND373, 354VPEEIM YSFASARAV 368, 357EIM YSFASARAV LPS361, 355PEEIM YSFASARAV L369, 356EEIM YSFASARAV LP370	I-Ab
GL50803_0015591	Coiled-coil protein	No	1595	176	111ILS LYASAALAS AVA125	I-Ab
GL50803_0017476	High cysteine membrane protein	Yes	2169	224	451VALF YTYTAANAV SS465, 452ALF YTYTAANAV SSY466, 449SKVALF YTYTAANAV 463, 454F YTYTAANAV SSYII468, 450KVALF YTYTAANAV S464, 453LF YTYTAANAV SSYI467	I-Ab

Peptide core is highlighted in grey. The polypeptide ID of conserved polypeptides between isolate WB and GS is red coloured. The pair of conserved polypeptides is denoted by a superscript number. *Homology with polypeptides of isolate WB does not present in the secretome. TM: transmembrane domains; MHC-II: major histocompatibility complex class II.

For the GS isolate, 10 polypeptides (3% of 350) were found to be part of the secretome (Table II). This group includes proteins such as cathepsin B, high-cysteine membrane proteins, and enzymes involved in the glycolysis pathway. As with the WB isolate, the peptide core sequence of the T-cell peptides is identical for all peptides derived from the same polypeptide.

**TABLE II:** Immunogenic polypeptides present in the secretome of *Giardia* assemblage B isolate GS

Polypeptide ID	Polypeptide name	TM domains	Polypeptide length	Molecular weight (kDa)	T-cell peptides	MHC-II
GL50581_4499	Hypothetical protein	No	281	30	76GVLV FKAAEPVAS ED90, 74SDGVLV FKAAEPVAS 88, 75DGVLV FKAAEPVAS E89, 77VLV FKAAEPVAS EDE91	I-Ab
GL50581_78 ^14^	Pept_C1 domain-containing protein	No	298	33	4FLLAA AAFSAPALT V18, 3LFLLAA AAFSAPALT 17, 8A AAFSAPALT VSELN22, 7AA AAFSAPALT VSEL21, 6LAA AAFSAPALT VSE20, 5LLAA AAFSAPALT VS19	I-Ab
GL50581_1446 ^2^	cAMP-dependent protein kinase regulatory chain	No	460	50	46IHK FASYSPLAA AVL60	I-Ab
GL50581_3080*	M20_dimer domain-containing protein	No	506	55	393VYES LKALASLAG FS407	I-Ad
GL50581_1032*	ANK_REP_REGION domain-containing protein	No	623	66	377AAI HAANAAAAT AND391, 376NAAI HAANAAAAT AN390, 378AI HAANAAAAT ANDN392, 375ANAAI HAANAAAAT A389	I-Ab
GL50581_3032	Kinase, NEK	No	782	86	517PVEP VYASAPVAL AE531, 518VEP VYASAPVAL AEL532	I-Ab
GL50581_411	High cysteine membrane protein Group 2	Yes	834	89	711AIDG YYYNSAKAS VT725, 713DG YYYNSAKAS VTQC727, 712IDG YYYNSAKAS VTQ726	I-Ab
GL50581_1272*	Protein 21.1	No	1032	113	663ET RAFAAACVN QMQE677, 661LEET RAFAAACVN QM675, 662EET RAFAAACVN QMQ676	I-Ab
GL50581_2252 ^3^	Glycerol-3-phosphate dehydrogenase	No	1111	119	356KEIM YSFASARAV LP370, 357EIM YSFASARAV LPC371, 355PKEIM YSFASARAV L369, 358IM YSFASARAV LPCN372, 354TPKEIM YSFASARAV 368, 359M YSFASARAV LPCND373	I-Ab
GL50581_881	Hypothetical protein	No	1904	214	1291YI RVYMGSTAA PAAA1305, 1292I RVYMGSTAA PAAAP1306	I-Ab

Peptide core is highlighted in grey. The polypeptide ID of conserved polypeptides between isolate WB and GS is red coloured. The pair of conserved polypeptides is denoted by a superscript number. *Homology with polypeptides of isolate WB does not present in the secretome. TM: transmembrane domains; MHC-II: major histocompatibility complex class II.

Notably, all immunogenic proteins identified in the secretomes of both *G. lamblia* isolates showed affinity for I-Ab. Additionally, only one protein from each isolate exhibited affinity for the I-Ad class II molecule.

*Specific interactions of the immunogenic T-cell peptides of G. lamblia* - MHC class II molecules consist of two polymorphic chains, α and β, which together form the peptide-binding groove. This groove interacts with a sequence of nine amino acids, known as the peptide core. The groove contains pockets that bind specific amino acids within the peptide core, thereby influencing the affinity of the MHC class II molecule for a particular T-cell peptide. The anchor residues for the I-Ad molecule are located in pockets P1, P4, and P9, with the model antigen corresponding to the OVA_323-339_ peptide (SQAVHAAHA). Pocket P1 is the least restrictive, accommodating amino acids of various sizes and charges with minimal impact on peptide-binding affinity. Pocket P4 is the most restrictive, preferentially accommodating small, uncharged amino acids. Pocket P9 is the second most restrictive and also favours small, uncharged amino acids. As shown in Table III, T-cell peptides from both isolates contain methionine in pocket P4 and alanine in pocket P9. For pocket P1, peptide 114-128 (VIKMSALPA) from the WB isolate contains valine, whereas peptide 194–208 (LKAMKAVAA) from the GS isolate contains leucine.

**TABLE III:** Interactions of immunogenic T-cell peptides of *Giardia* isolates WB and GS with the binding groove of MHC-II molecules

		Peptide core									IC_50_ (nM)
		P1	P2	P3	P4	P5	P6	P7	P8	P9	
I-A^d^	OVA (323-339)	S	Q	A	V	H	A	A	H	A	
	WB (114-128)	V	I	K	M	S	A	L	P	A	5.2
		GS (194-208)	L	K	A	M	K	A	V	A	A	6.5
I-E^k^	Hsp70 (236-248)	V	N	H	F	I	A	E	F	K	
	WB (375-389)	V	I	R	M	I	Y	F	Y	K	7.8
	GS (373-387)	V	I	R	M	I	Y	F	Y	K	7.8
I-A^k^	HEL (50-62)	D	Y	G	I	L	Q	I	N	S	
	WB (204-218)	H	Q	D	Y	N	Q	N	Q	I	523.2
	GS (125-139)	N	A	H	S	A	H	S	N	H	566.6

MHC-II: major histocompatibility complex class II.

The I-Ek molecule has a peptide-binding groove with anchor residues located in pockets P1, P4, P6, and P9. Pockets P1 and P9 are the most restrictive. Pocket P1 accommodates small hydrophobic amino acids such as valine, leucine, and isoleucine, while pocket P9 is suited to large hydrophilic amino acids, preferentially lysine or arginine. Hydrophobic amino acids predominate in pocket P4, whereas charged amino acids are common in pocket P6. As shown in Table III, T-cell peptides from both isolates, GS375-389 (VIRMIYFYK) and WB373-387 (VIRMIYFYK), contain valine at P1 and lysine at P9, consistent with the model antigen Hsp70_236-248_ (VNHFIAEFK).

The peptide-binding groove of the I-Ak molecule features anchor residues in pockets P1, P4, P6, and P9. Pocket P1 is the most restrictive, accommodating negatively charged amino acids, with a preference for aspartic acid. Pocket P6 is the second most restrictive, preferentially accommodating glutamic acid and glutamine. Although pocket P4 is less restrictive, it preferentially accommodates medium-sized hydrophobic amino acids such as isoleucine, valine, and leucine. Pocket P9 is the least restrictive, allowing a variety of residues to fit. As shown in Table III, the model antigen peptide (hen egg white lysozyme, HEL_50-62_, DYGILQINS) presents aspartic acid in pocket P1, whereas the peptide from the WB isolate contains histidine and that from the GS isolate contains asparagine. For pocket P4, HEL exhibits isoleucine, while the T-cell peptides from the WB and GS isolates present tyrosine and serine, respectively. The model antigen shows glutamine in pocket P6, as does the peptide from WB, whereas the peptide from GS contains histidine. Finally, for pocket P9, the peptide from WB contains isoleucine, whereas the peptide from GS contains histidine.

## DISCUSSION

In the present study, we performed an immunoinformatic analysis of the entire proteome of *G. lamblia* assemblages A and B to identify potential immunogenic antigens. Notably, in both assemblages, most polypeptides exhibited higher affinity for I-Ab and I-Ek molecules, emphasising the influence of the structural characteristics of each MHC class II binding groove. The results shown in Figure indicate that immunogenic polypeptides bind to MHC class II molecules in different ways. The I-Ek allele exhibited higher affinity for most polypeptides, whereas the I-Ak allele did not show high affinity for any of the polypeptides analysed. This is likely due to the distinct structural requirements of the binding groove of each MHC class II molecule. The I-Ak molecule possesses one of the most restrictive binding grooves, with the principal constraint being the requirement for peptides to contain a negatively charged residue — primarily aspartic acid — at the P1 position of the core, in order to form stable, long-lasting interactions with the I-Ak molecule.^21^ ^,^ ^23^ This effect is illustrated in Table III, which shows the interactions of T-cell peptides with the highest affinity for MHC class II molecules (I-Ad, I-Ek, and I-Ak) from both WB and GS isolates. The low affinity of peptides for I-Ak molecules may be due to their inability to fit properly into the binding groove, resulting from the absence of negatively charged amino acids, such as aspartic acid or glutamic acid.

The I-Ek allele was the MHC class II molecule that bound the greatest number of polypeptides and T-cell peptides from both isolates (Figure A and C). Several studies report that most of the peptides eluted from I-Ek contain hydrophobic amino acids — primarily valine, leucine, and isoleucine — at pocket P1. Nevertheless, other residues with hydrophobic properties can also occupy this pocket.^24^ ^,^ ^25^ ^,^ ^26^ Most polypeptides from both isolates contain T-cell peptides with hydrophobic amino acids at the P1 position, which may explain why a larger number of polypeptides exhibited higher affinity for I-Ek compared with the other MHC class II molecules analysed in this study.

An ideal vaccine antigen should confer cross-protection against different strains from the two genetic assemblages of *G. lamblia* that cause human disease. In this study, we analysed conserved immunogenic polypeptides between assemblages A and B and identified several highly conserved polypeptides with greater than 80% homology [Supplementary data (Table I)]. Among these, some proteins — such as dyneins and NEK kinase — are considered constitutive, as they are expressed throughout all stages of the parasite’s cell cycle. Additionally, several of the identified polypeptides correspond to molecules that perform key biological functions in *Giardia* or act as virulence factors. Some of these conserved polypeptides belong to the family of proteases known as cathepsins, with cathepsin B-like proteases being the most highly expressed in *Giardia*.^27^

Only a few complete epitopes were found to be conserved among the polypeptides of the assemblages analysed in this study. However, we identified numerous peptide cores that were highly conserved within the corresponding immunogenic polypeptides of *Giardia*. These highly conserved peptide cores appear to be minimally affected by parasite adaptation and immune evasion, or they may form part of functionally important domains of the polypeptides.

Promiscuity can enhance the host immune response by increasing the likelihood that a polypeptide will be presented to T cells. In this study, we defined promiscuous polypeptides as those generating T-cell peptides capable of binding to more than one MHC class II molecule. We identified 21 promiscuous polypeptides in the WB isolate [Supplementary data (Table II)] that can bind to different MHC class II molecules, whereas 16 promiscuous polypeptides were found in the GS isolate [Supplementary data (Table III)]. Although MHC molecules are highly polymorphic, H2 shares key conserved regions with human HLA antigens.^28^ It would be of interest to investigate whether these polypeptides can be recognised by one or more human HLA molecules. Previous studies have reported that individuals infected with *Giardia* express the HLA haplotypes HLA-DRB103:01, HLA-DRB113:01, and HLA-DRB107:01. In contrast, individuals expressing HLA-DRB104:02, HLA-DRB1 10:01, HLA-DRB114:01, and HLA-DRB1*15:01 appear to be resistant to the parasite.^6^ ^,^ ^29^ ^,^ ^30^ This is particularly noteworthy because the peptide-binding groove of I-Ek has been reported to be highly similar to that of HLA-DR molecules.^20^

*Giardia lamblia* is a luminal parasite, meaning that it cannot cross the epithelial barrier. This raises questions regarding the mechanisms employed by the protozoan to elicit an immune response. The study of the excretory-secretory products (ESPs) of *G. lamblia*, collectively referred to as the *Giardia* secretome,^31^ may help to elucidate how this parasite stimulates host immune responses. Among the various secreted molecules, cysteine proteases (CPs) are recognised as major virulence factors and play a critical role during infection. Our results indicate that this group of proteins is highly immunogenic and is present in the secretome of both isolates (Tables I-II). In addition to previous studies emphasising their role in virulence during infection, these proteins may serve as potential targets for the development of anti-*Giardia* therapies^32^ or as tools to improve our understanding of the parasite’s behaviour and its interaction with the host immune system.

High-cysteine membrane proteins (HCMPs) have also been identified as components of *Giardia* ESPs and represent the most abundant differentially expressed genes when *Giardia* interacts with intestinal epithelial cells.^33^ ^,^ ^34^

Assemblages A and B are responsible for human giardiasis; therefore, antigens conserved across both assemblages, as well as those present in the *Giardia* secretome, represent ideal vaccine candidates. In this study, we identified all potential immunogenic antigens of this parasite using immunoinformatics. Among these, six polypeptides were conserved in both assemblages and present in the secretome: cathepsin B (GL50803_0016779), protein kinase regulatory chain (GL50803_009117), glycerol-3-phosphate dehydrogenase (GL50803_0016125), M_20 dimer domain-containing protein (GL50581_3080), ANK_REP_REGION domain-containing protein (GL50581_1032), and protein 21.1 (GL50581_1272). Two to three T-cell peptides from each of these polypeptides could be incorporated into a multiepitope vaccine against *Giardia*. Multiepitope vaccine technology has been widely applied in the design and development of vaccines against a range of pathogens, including dengue virus, HIV-1, influenza virus, and *Leishmania*,^35^ ^,^ ^36^ ^,^ ^37^ ^,^ ^38^ making it a promising strategy for giardiasis vaccine design. While GiardiaDB contains some unvalidated entries, which should be considered when selecting candidate antigens, the selected candidates display the key features of immunogenic proteins, emphasising their strong potential for inclusion in a vaccine.

In the present study, we conducted an immunoinformatic analysis to identify immunogenic polypeptides in *G. lamblia* assemblages A and B. While immunoinformatic tools are invaluable for integrating large-scale immunological data in a short period, thereby facilitating drug and vaccine development, several limitations remain to be considered. Based on our final cut-off criteria, we did not identify polypeptides corresponding to proteins previously described as immunogenic, such as CWPs, giardins, and tubulins. Nevertheless, initial analyses using less stringent thresholds indicated that several polypeptides from CWPs, VSPs, giardins, and metabolic proteins contained MHC class II-restricted peptides with percentile ranks of 0.1% or 1%, which are still considered strong binders. These proteins have been experimentally confirmed as immunogenic.^6^ Furthermore, we evaluated the selected polypeptides across multiple threshold combinations [Supplementary data (Tables IV-V)]. Collectively, these findings underscore the robustness and feasibility of our approach for identifying immunogenic candidates. Additionally, our analysis revealed that none of the *Giardia* polypeptides were predicted to be immunogenic for I-Ak under the parameters selected, despite experimental evidence from our research group demonstrating the immunogenic activity of *Giardia* antigens for I-Ak.^39^ ^,^ ^40^ ^,^ ^41^ Although experimentally validated *Giardia* T-cell epitopes are not yet available, our predictions provide a valuable foundation for future laboratory validation, thereby advancing the identification of potential vaccine targets. These findings emphasise the limitations of immunoinformatic tools in capturing all aspects of immunogenicity during rapid, large-scale epitope screening. For MHC class II prediction servers, it would be advantageous to additionally consider factors such as antigen processing, the flanking amino acid sequences, interactions with the T-cell receptor, and the potential effector mechanisms of T cells. Moreover, during the processing of exogenous antigens, HLA-DM accessory molecules play a critical role in the selection of immunodominant epitopes and their restricted presentation by MHC class II molecules.^42^ ^,^ ^43^ Despite advances in T-cell epitope prediction through *in silico* approaches, there remains a need for improved tools capable of accurately identifying immunogenic MHC class II epitopes. Such tools should incorporate additional determinant features — such as those described above — to enhance the precision of epitope screening.

The limitations outlined previously underscore the importance of validating MHC class II predictions *in vivo* to confirm the true immunogenicity of the selected polypeptides. In this context, the value of performing MHC class II predictions using murine alleles becomes apparent. This strategy allows for *in vivo* testing of predicted immunogenicity, as the selected murine MHC class II alleles correspond to some of the most commonly used mouse strains in *Giardia* research: C57BL/6 (haplotype b), BALB/c (haplotype d), and C3H/HeJ (haplotype k). With regard to experimental validation, the ideal next step would be to select a subset of polypeptides and perform analyses in one or more of the murine models mentioned above to confirm immunogenicity. The resulting data will inform the potential use of these polypeptides in future studies involving human models.

Another limitation of our study is the reliance on GiardiaDB as the source of *Giardia* proteomes. Like other biological databases (*e.g.*, GenBank, IEDB, UniProt), GiardiaDB contains protein sequences that are computational predictions and may include redundancies or unvalidated entries. Consequently, some of the immunogenic polypeptides identified may not correspond to bona fide *Giardia* proteins. To our knowledge, this is the first study to analyse the complete proteome of *Giardia* with the aim of identifying all its potential immunogenic antigens. This knowledge will contribute to the future development of novel prophylactic approaches against giardiasis and pave the way for implementing a combinatorial strategy that integrates immunoinformatics and data science to investigate other pathogens beyond *G. lamblia*.

## SUPPLEMENTARY MATERIALS

Supplementary material

## Data Availability

The data that support the findings of this study are openly available in GitHub at https://github.com/davidortega47-rgb/Datasets_Immunoinformatic_Prediction_G.lamblia.git and https://github.com/davidortega47-rgb/LIBCE-codes.git. Also, these data can be found in Zenodo at https://doi.org/10.5281/zenodo.18210685 and https://doi.org/10.5281/zenodo.18166880. Supplementary data associated with this article can be found in the online version.
