# Supplementary material for: Immunoinformatic analysis for identifying immunogenic antigens from the complete proteome of Giardia lamblia
Source: Mem Inst Oswaldo Cruz. 2026 Apr 17;121:e250216. doi: 10.1590/0074-02760250216 (PMC13090037; doi:10.1590/0074-02760250216)
Supplement: Supplementary material [file 1678-8060-mioc-121-e250216-s1.pdf]

TABLE I

Conserved immunogenic polypeptides between *Giardia* assemblages A and B

Giardia assemblage A isolate WB			*Giardia* assemblage B isolate GS			
Polypeptide ID	Polypeptide name	T-cell peptides	Polypeptide ID	Polypeptide name	T-cell peptides	Homology (%)
GL50803_0016804B	Outer-arm dynein gamma	$_{908}$CVAHILRSRIIRMP$_{922}$<br>$_{907}$ACVAHILRSRIIRM$_{921}$<br>$_{1213}$TKANEMMKVISVSTA$_{1227}$<br>$_{1216}$NEMMKVISVSTAEAE$_{1230}$<br>$_{1214}$KANEMMKVISVSTAE$_{1228}$<br>$_{1215}$ANEMMKVISVSTAEA$_{1229}$	GL50581_2837	Dynein heavy chain	$_{1212}$TKANEMMKVISVSTA$_{1226}$<br>$_{906}$ACVAHILRSRIIRM$_{920}$<br>$_{1215}$NEMMKVISVSTAEAE$_{1229}$<br>$_{907}$CVAHILRSRIIRMP921<br>$_{1213}$KANEMMKVISVSTAE$_{1227}$<br>$_{1214}$ANEMMKVISVSTAEA$_{1228}$	99
GL50803_008001	Ribosomal protein L15	$_{56}$KRGYVVYRVRVRRGG$_{70}$<br>$_{57}$RGYVVYRVRVRRGGR$_{71}$<br>$_{59}$YVVYRVRVRRGGRKR$_{73}$<br>$_{58}$GYVVYRVRVRRGGRK$_{72}$	GL50581_979	Ribosomal protein L15	$_{58}$GYVVYRVRVRRGGRK$_{72}$<br>$_{59}$YVVYRVRVRRGGRKR$_{73}$<br>$_{57}$RGYVVYRVRVRRGGR$_{71}$<br>$_{56}$KRGYVVYRVRVRRGG$_{70}$	99
GL50803_0012163	Unspecified product	$_{200}$KQKKTMLAALAASER$_{214}$<br>$_{201}$QKKTMLAALAASERT$_{215}$	GL50581_1069	Dynein regulatory complex protein 10	$_{200}$KQKKTMLAALAASER$_{214}$<br>$_{201}$QKKTMLAALAASERT$_{215}$	98
GL50803_005786	Putative Ribosome biogenesis protein	$_{204}$ACISQYALSAAAVAA$_{218}$<br>$_{206}$ISQYALSAAAVAARV$_{220}$<br>$_{205}$CISQYALSAAAVAAR$_{219}$	GL50581_4082	Ribosome biogenesis protein NEP1, putative	$_{205}$CISQYALSAAAVAAR$_{219}$<br>$_{206}$ISQYALSAAAVAARV$_{220}$<br>$_{204}$ACISQYALSAAAVAA$_{218}$	98
GL50803_0017547	Ribosomal protein L4	$_{109}$QRRYAVASALAASAN$_{123}$<br>$_{110}$RRYAVASALAASANA$_{124}$<br>$_{111}$RYAVASALAASANAA$_{125}$<br>$_{108}$NQRRYAVASALAASA$_{122}$<br>$_{107}$LNQRRYAVASALAAS$_{121}$	GL50581_4247	Ribos_L4_asso_C domain-containing protein	$_{109}$QRRYAVASALAASAN$_{123}$<br>$_{110}$RRYAVASALAASANA$_{124}$<br>$_{107}$LNQRRYAVASALAAS$_{121}$<br>$_{108}$NQRRYAVASALAASA$_{122}$<br>$_{111}$RYAVASALAASANAS$_{125}$	98
GL50803_001695	Rab11	$_{28}$FTSNKFNASSAATIG$_{42}$<br>$_{27}$RFTSNKFNASSAATI$_{41}$<br>$_{29}$TSNKFNASSAATIGV$_{43}$	GL50581_3346	Rab11	$_{28}$FTSNKFNASSAATIG$_{42}$<br>$_{29}$TSNKFNASSAATIGV$_{43}$<br>$_{27}$RFTSNKFNASSAATI$_{41}$	98
GL50803_005744	Sec61 alpha family protein	$_{284}$RLLYASSTPMMIIST$_{298}$<br>$_{281}$YPIRLLYASSTPMMI$_{295}$<br>$_{282}$PIRLLYASSTPMMII$_{296}$<br>$_{283}$IRLLYASSTPMMIIS$_{297}$	GL50581_1683	Plug_translocon domain-containing protein	$_{283}$IRLLYASSTPMMIIS$_{297}$<br>$_{282}$PIRLLYASSTPMMII$_{292}$<br>$_{281}$YPIRLLYASSTPMMI$_{285}$<br>$_{284}$RLLYASSTPMMIIST$_{298}$	97
GL50803_0011311	Kinase, NEK	$_{294}$IEMNERIKAMPAYAA$_{308}$<br>$_{297}$NERIKAMPAYAAQAP$_{311}$<br>$_{298}$ERIKAMPAYAAQAPP$_{312}$<br>$_{296}$MNERIKAMPAYAAQA$_{310}$<br>$_{295}$EMNERIKAMPAYAAQ$_{309}$<br>$_{299}$RIKAMPAYAAQAPPE$_{313}$	GL50581_3516	Protein kinase domain-containing protein	$_{297}$NERIKAMPAYAAQAP$_{311}$<br>$_{298}$ERIKAMPAYAAQAPP$_{312}$<br>$_{296}$MNERIKAMPAYAAQA$_{310}$<br>$_{295}$EMNERIKAMPAYAAQ$_{309}$<br>$_{294}$IEMNERIKAMPAYAA$_{308}$<br>$_{299}$RIKAMPAYAAQAPPE$_{313}$	97
GL50803_0017060	Ankyrin repeat protein 1	$_{375}$ANAALHAANAAAAAA$_{389}$<br>$_{377}$AALHAANAAAAAATD$_{391}$<br>$_{376}$NAALHAANAAAAAAT$_{390}$<br>$_{378}$ALHAANAAAAAATDK$_{392}$<br>$_{374}$RANAALHAANAAAAA$_{388}$	GL50581_1032	ANK_REP_REGION domain-containing protein	$_{377}$AAIHAANAAAATAND$_{391}$<br>$_{376}$NAAIHAANAAAATAN$_{390}$<br>$_{378}$AIHAANAAAATANDN$_{392}$<br>$_{375}$ANAAIHAANAAAATA$_{389}$	97
GL50803_0015218	WD40 repeat protein	$_{390}$SSSAAFTSAATSAVS$_{404}$<br>$_{391}$SSAAFTSAATSAVSS$_{405}$<br>$_{389}$VSSSAAFTSAATSAV$_{403}$	GL50581_2535	WD_REPEATS_ REGION domain-containing protein	$_{381}$VSSSAAFTSAATSAV$_{395}$<br>$_{383}$SSAAFTSAATSAVSS$_{397}$<br>$_{382}$SSSAAFTSAATSAVS$_{396}$	96
GL50803_009909	Pyruvate, phosphate dikinase	$_{869}$ARVAAAIAAIKARTN$_{883}$<br>$_{868}$VARVAAAIAAIKART$_{882}$	GL50581_4299	Pyruvate, phosphate dikinase	$_{868}$VARVAAAIAAIKART$_{882}$<br>$_{869}$ARVAAAIAAIKARTN$_{883}$	96
GL50803_0015120	Hypothetical protein	$_{136}$LKTAYKFTPALPKTI$_{150}$	GL50581_233	unspecified product	$_{136}$LKKAYKFTPALPRTI$_{150}$<br>$_{137}$KKAYKFTPALPRTIH$_{151}$	96
GL50803_00112076	Kinase, CDC7	$_{1185}$MLAQAVAIKSLAAKY$_{1199}$<br>$_{1183}$PSMLAQAVAIKSLAAI$_{197}$<br>$_{1186}$LAQAVAIKSLAAKYA$_{1200}$<br>$_{1184}$SMLAQAVAIKSLAAK$_{1198}$	GL50581_2723	Protein kinase domain-containing protein	$_{1186}$LAQAVAIKSLAAKYA$_{1200}$<br>$_{1185}$MLAQAVAIKSLAAKY$_{1199}$<br>$_{1183}$PSMLAQAVAIKSLAA$_{1197}$<br>$_{1184}$SMLAQAVAIKSLAAK$_{1198}$	96
GL50803_0033592	unspecified product	$_{777}$YISWRRSIRMRAGDE$_{793}$<br>$_{775}$EGAHYISWRRSIRMR$_{789}$<br>$_{778}$HYISWRRSIRMRAGD$_{792}$<br>$_{776}$GAHYISWRRSIRMRA$_{790}$<br>$_{777}$AHYISWRRSIRMRAG$_{791}$	GL50581_3534	Hypothetical protein	$_{777}$SHYVSWRRSIRVRAG$_{791}$<br>$_{776}$GSHYVSWRRSIRVRA$_{790}$<br>$_{778}$HYVSWRRSIRVRAGD$_{792}$<br>$_{775}$EGSHYVSWRRSIRVR$_{789}$<br>$_{779}$YVSWRRSIRVRAGDE$_{793}$	95
GL50803_007865	L-asparaginase	$_{385}$RGEIETFQASTARSN$_{399}$<br>$_{386}$GEIETFQASTARSNY$_{400}$	GL50581_1474	Asparaginase	$_{385}$RGEIETFQASTARSN$_{399}$<br>$_{386}$GEIETFQASTARSNY$_{400}$	95

Peptide core is highlighted in red.

TABLE II

Promiscuous polypeptides in *Giardia* assemblage A isolate WB

Polypeptide ID	Polypeptide name	TM domains	Polypeptide length	Molecular weight (kDa)	T-cell peptide core	MHC-II
GL50803_005273	Unspecified product	No	360	41	[1,2]YYAMSPLSA	[1]I-A^{b}, [2]I-A^{d}
GL50803_0010516	Leucine carboxyl methyltransferase	No	364	41	[1]SFWPARRRE, [2]LSKLSALRA	[1]I-E^{d}, [2]I-A^{d}
GL50803_0022291	EamA-like transporter family protein	Yes	472	52	[1]FLRRRVRLY, [2]LIYFVSILF	[1]I-E^{d}, [2]I-E^{k}
GL50803_0011897	Putative Phosphatidylinositol-4-phosphate 5-kinase	No	506	57	[1]YAFSPACAL, [2]VFRRRFTFE	[1]I-A^{b}, [2]I-E^{d}
GL50803_0014727	Unspecified product	No	572	64	[1]IIRFYIWMW, [2]LRARASISA	[1]I-E^{k}, [2]I-A^{d}
GL50803_0061047	Amino acid permease	Yes	673	74	[1]LIVLYFGFL, [1]IVLYFGFLT, [2]TKVLSAIAS	[1]I-E^{k}, [2]I-A^{d}
GL50803_0091712	Amino acid transporter family	Yes	707	77	[1]LIVLYFGFL, [1]IVLYFGFLT, [2]TKVLSAIAS	[1]I-E^{k}, [2]I-A^{d}
GL50803_009573	Unspecified product	No	769	87	[1]YYVNSPVAS, [2]IIHMLRYLV	[1]I-A^{b}, [2]I-E^{k}
GL50803_0024133	5'-3' exoribonuclease 2	No	902	103	[1]YFYKQLKRS, [2]LIWVFRYYF, [2]VIRMIYFYK	[1]I-E^{d}, [2]I-E^{k}
GL50803_003342	Unspecified product	Yes	1002	113	[1]LIVHVLYLV, [1]IVHVLYLVH, [2]IQHLAALSS	[1]I-E^{k}, [2]I-A^{d}
GL50803_00137731	CDC45-like protein	No	1043	117	[1]YMKLRAIAS, [2]LFYRQRYRK	[1]I-A^{d}, [2]I-E^{d}
GL50803_0016715	Unspecified product	No	1090	122	[1]TAAILALKA, [1]ILALKALSS, [2]MIRVIRFLF, [2]VIRFLFMRI	[1]I-A^{d}, [2]I-E^{k}
GL50803_0016958	Putative Phospholipid-transporting ATPase IA	Yes	1284	144	[1]LVTFIRWII, [1]FVRFLYVYF, [2]NYFYRYRIH, [2]YFYRYRIHP	[1]I-E^{k}, [2]I-E^{d}
GL50803_0024861	Unspecified product	No	1300	145	[1]ANYSATLAA, [2]MRQIAALKA	[1]I-A^{b}, [2]I-A^{d}
GL50803_0014058	CAMP-specific 3',5'-cyclic phosphodiesterase 4B	Yes	1371	155	[1]IIVYICAIK, [1]YICAIKFVY, [2]YFYRQRPSY	[1]I-E^{k}, [2]I-E^{d}
GL50803_0017006	Unspecified product	No	1381	164	[1]RFYRLIRRW, [1]RLIRRWKRT [2]LLRHAFMIW, [1]FWTWARRLR	[1]I-E^{d}, [2]I-E^{k}
GL50803_0017439	Unspecified product	No	1918	217	[1]WQSVQALAV, [2]LLHYFRFFY	[1]I-A^{d}, [2]I-E^{k}
GL50803_00102515	Putative RNA-directed RNA polymerase	No	2001	225	[1]VILYYLFYT, [2]KMSLSAIAS	[1]I-E^{k}, [2]I-A^{d}
GL50803_0060562	Unspecified product	No	2109	234	[1]LVYYIKRFA, [2]AFWRKYTYY, [1]MIYYYRLLF	[1]I-E^{k}, [2]I-E^{d}
GL50803_0016804B	Outer-arm dynein gamma	No	2676	302	[1]HILRISRII, [2]MMKVISVST	[1]I-E^{k}, [2]I-A^{d}
GL50803_0016492	Unspecified product	Yes	2915	328	[1]ISKISSISA, [2]YFVKRVRRV	[1]I-A^{d}, [2]I-E^{d}

TM: transmembrane domains; MHC-II: major histocompatibility complex class II.

TABLE III

Promiscuous polypeptides in *Giardia* assemblage B isolate GS

Polypeptide ID	Polypeptide name	TM domains	Polypeptide length	Molecular weight (kDa)	T-cell peptide core	MHC-II
GL50581_4284	Hypothetical protein	Yes	157	18	[1]FQFKSALAT, [2]IITVVRYFI	[1]I-A^{b}, [2]I-E^{k}
GL50581_515	Hypothetical protein	Yes	237	26	[1]MFIRKRKSS, [2]LIIYFMFIR	[1]I-E^{d}, [2]I-E^{k}
GL50581_1825	Leucine carboxyl methyltransferase 1	No	367	42	[1]LSKLSALRA, [2]SFWPARRRE	[1]I-A^{d}, [2]I-E^{d}
GL50581_2773	EamA domain-containing protein	Yes	479	53	[1]VIYFISILF, [1]LVFVVIYFI, [2]FLRRRVRLY	[1]I-E^{k}, [2]I-E^{d}
GL50581_399	DinF protein	Yes	573	65	[1]VIYLIRLVV, [2]RYVWQWKVY	[1]I-E^{k}, [2]I-E^{d}
GL50581_1246	Hypothetical protein	Yes	611	68	[1]VISMKTIRI, [2]IWFRIRRYL	[1]I-A^{d}, [2]I-E^{d}
GL50581_1488	Hypothetical protein	Yes	756	85	[1]YIKYFRSFL, [2]MAQMSAIPI	[1]I-E^{k}, [2]I-A^{d}
GL50581_4134	Hypothetical protein	Yes	823	92	[1]RKQVVALRA, [2]YWRRTYKAY	[1]I-A^{d}, [2]I-E^{d}
GL50581_2730	5'-3' exoribonuclease 2	No	900	103	[1]VIRMIYFYK, [2]YFYKQLKRS	[1]I-E^{k}, [2]I-E^{d}
GL50581_932	Hypothetical protein	No	1159	129	[1]AYYRMFRAR, [2]IIRYITFAL	[1]I-E^{d}, [2]I-E^{k}
GL50581_900	Phospholipid-transporting ATPase IIB, putative	Yes	1432	159	[1]YVIFFRYLL, [1]VIFFRYLLL, [2]FFLRWIRMR	[1]I-E^{k}, [2]I-E^{d}
GL50581_4217	Hypothetical protein	No	1891	209	[1]YLAVVYSVR, [1]VVYSVRLFR, [2]YSFAVPTVT	[1]I-E^{k}, [2]I-A^{b}
GL50581_1757	ABC transporter, ATP-binding protein, putative	Yes	2071	232	[1]YIFYSPALS, [2]FLVHLGFCF	[1]I-A^{b}, [2]I-E^{k}
GL50581_4510	Hypothetical protein	No	2112	235	[1]AFWKKYTYY, [2]LVYYIKRFA	[1]I-E^{d}, [2]I-E^{k}
GL50581_2118	Hypothetical protein	No	2119	239	[1]RYFRVLRAS, [1]LWLRYFRVL, [2]WLRYFRVLR, [2]FMLWLRYFR, [2]LIRMIFMLW	[1]I-E^{d}, [2]I-E^{k}
GL50581_2837	Dynein heavy chain	No	2675	302	[1]MMKVISVST, [2]HILRISRII	[1]I-A^{d}, [2]I-E^{k}

TM: transmembrane domains; MHC-II: major histocompatibility complex class II.

TABLE IV

Prediction of immunogenic polypeptides at different thresholds in *Giardia* assemblage B isolate GS

	PR ≤ 0.01, IC$_{50}$ 50 nM	PR ≤ 0.1, IC$_{50}$ 50 nM	PR ≤ 1.0, IC$_{50}$ 50 nM	*Total	**Overlapping polypeptides
Number of polypeptides	350	1682	2870	4902	2032
Number of promiscuous polypeptides	16	427	1281	1724	1281

PR: percentile rank; *total number of polypeptides predicted across all three thresholds; **polypeptides overlapping across the three thresholds.

TABLE V

Prediction of immunogenic polypeptides at different thresholds in *Giardia* assemblage A isolate WB

	PR ≤ 0.01, IC$_{50}$ 50 nM	PR ≤0.1, IC$_{50}$ 50 nM	PR ≤ 1.0, IC$_{50}$ 50 nM	*Total	**Overlapping polypeptides
Number of polypeptides	414	1887	3195	5496	2301
Number of promiscous polypeptides	21	507	1412	1940	1412

PR: percentile rank; *total number of polypeptides predicted across all three thresholds; **polypeptides overlapping across the three thresholds.
